# Improving the Prediction of Benign or Malignant Breast Masses Using a Combination of Image Biomarkers and Clinical Parameters

**DOI:** 10.3389/fonc.2021.629321

**Published:** 2021-03-22

**Authors:** Yanhua Cui, Yun Li, Dong Xing, Tong Bai, Jiwen Dong, Jian Zhu

**Affiliations:** ^1^Department of Radiation Oncology Physics and Technology, Shandong Cancer Hospital and Institute, Shandong First Medical University and Shandong Academy of Medical Sciences, Jinan, China; ^2^Department of Radiology, Shandong Cancer Hospital and Institute, Shandong First Medical University and Shandong Academy of Medical Sciences, Jinan, China; ^3^Department of Radiology, Yantai Yuhuangding Hospital, Yantai, China; ^4^Shandong Provincial Key Laboratory of Network based Intelligent Computing, School of Information Science and Engineering, University of Jinan, Jinan, China; ^5^Shandong Medical Imaging and Radiotherapy Engineering Technology Research Center, Jinan, China; ^6^Shandong College Collaborative Innovation Center of Digital Medicine Clinical Treatment and Nutrition Health, Qingdao, China; ^7^Shandong Provincial Key Laboratory of Digital Medicine and Computer-Assisted Surgery, Qingdao, China

**Keywords:** mammography, image feature, deep learning, clinical prediction, radiomics

## Abstract

**Background:** Breast cancer is one of the leading causes of death in female cancer patients. The disease can be detected early using Mammography, an effective X-ray imaging technology. The most important step in mammography is the classification of mammogram patches as benign or malignant. Classically, benign or malignant breast tumors are diagnosed by radiologists' interpretation of mammograms based on clinical parameters. However, because masses are heterogeneous, clinical parameters supply limited information on mammography mass. Therefore, this study aimed to predict benign or malignant breast masses using a combination of image biomarkers and clinical parameters.

**Methods:** We trained a deep learning (DL) fusion network of VGG16 and Inception-V3 network in 5,996 mammography images from the training cohort; DL features were extracted from the second fully connected layer of the DL fusion network. We then developed a combined model incorporating DL features, hand-crafted features, and clinical parameters to predict benign or malignant breast masses. The prediction performance was compared between clinical parameters and the combination of the above features. The strengths of the clinical model and the combined model were subsequently validated in a test cohort (*n* = 244) and an external validation cohort (*n* = 100), respectively.

**Results:** Extracted features comprised 30 hand-crafted features, 27 DL features, and 5 clinical features (shape, margin type, breast composition, age, mass size). The model combining the three feature types yielded the best performance in predicting benign or malignant masses (AUC = 0.961) in the test cohort. A significant difference in the predictive performance between the combined model and the clinical model was observed in an independent external validation cohort (AUC: 0.973 vs. 0.911, p = 0.019).

**Conclusion:** The prediction of benign or malignant breast masses improves when image biomarkers and clinical parameters are combined; the combined model was more robust than clinical parameters alone.

## Introduction

Breast cancer is one of the leading causes of death in female cancer patients. Early diagnosis of the condition is crucial to improve the survival rate and relieve suffering in patients (1). Mammography is an effective X-ray imaging technology that detects breast cancer early. Classically, benign or malignant breast tumors are diagnosed by radiologists' interpretation of mammograms based on clinical parameters. However, because masses are heterogeneous, clinical parameters supply limited information on mammography mass (2). There is, therefore, an urgent need to find new tools that can identify patients with breast cancer.

Machine learning (3) from artificial intelligence (AI) has made progress in automatically quantifying the characteristics of masses (4). Radiomics is an emerging field in quantitative imaging; it is a method that uses machine learning to transform images into high-dimensional and minable feature data (5, 6). With radiomics, clinical decision support can be improved. Exploratory research using this method has shown great promise in the diagnosis of breast masses (7). Radiomics can quantify large-scale information extracted from mammography images, which makes it a tool with better diagnostic capabilities for benign and malignant breast masses, and this method also provides radiologists with supplementary data (8). Analysis by radiomics requires machine learning methods with high levels of robustness and statistical power. This extraction method continues to be developed to improve its performance in evaluating masses, and this improvement, in turn, assists radiologists in accurately interpreting mammography imaging.

Hand-crafted-based radiomics extracts low-level features (texture features and shape features) as image biomarkers and estimate the likelihood of malignant masses based on extracted image biomarkers (9–11). In recent years, there has been significant progress on the subject of deep learning (12) (DL) and computer vision, with DL radiomics attaining remarkable heights in various medical imaging applications (13–15); DL directly learns unintuitive hidden features from images. DL features acquire more information and superior performance than hand-crafted image features (16). DL has only been used in a few studies in the field of mammography automatic diagnosis (17). Classifying benign or malignant masses, as compared to normal and abnormal areas, for the lack of obvious features is more complex. With the shift from hand-crafted to DL-based radiomics, combining deep learning and hand-crafted features have become more popular in radiomics most recently (18, 19).

In this study, we explore a DL fusion network of two different transfer-learning models combined with data augmentation, aimed at improving the classification accuracy. We hypothesized that image biomarkers (DL features and hand-crafted features) and clinical parameters could express intrinsic information on mass thoroughly when combined. We built a classification model that combines image biomarkers with clinical parameters and called it a combined model. Using clinical characteristics as the diagnostic information from mammography, we sought to determine the classification performance of the combined model and its clinical predictor. We evaluated predictive performance in two validation cohorts: the absence of mammography in the training cohort as the test cohort (inner-validation) and mammography from other hospitals as the external validation cohort.

## Materials and Methods

### Patients

**Figure 1:**
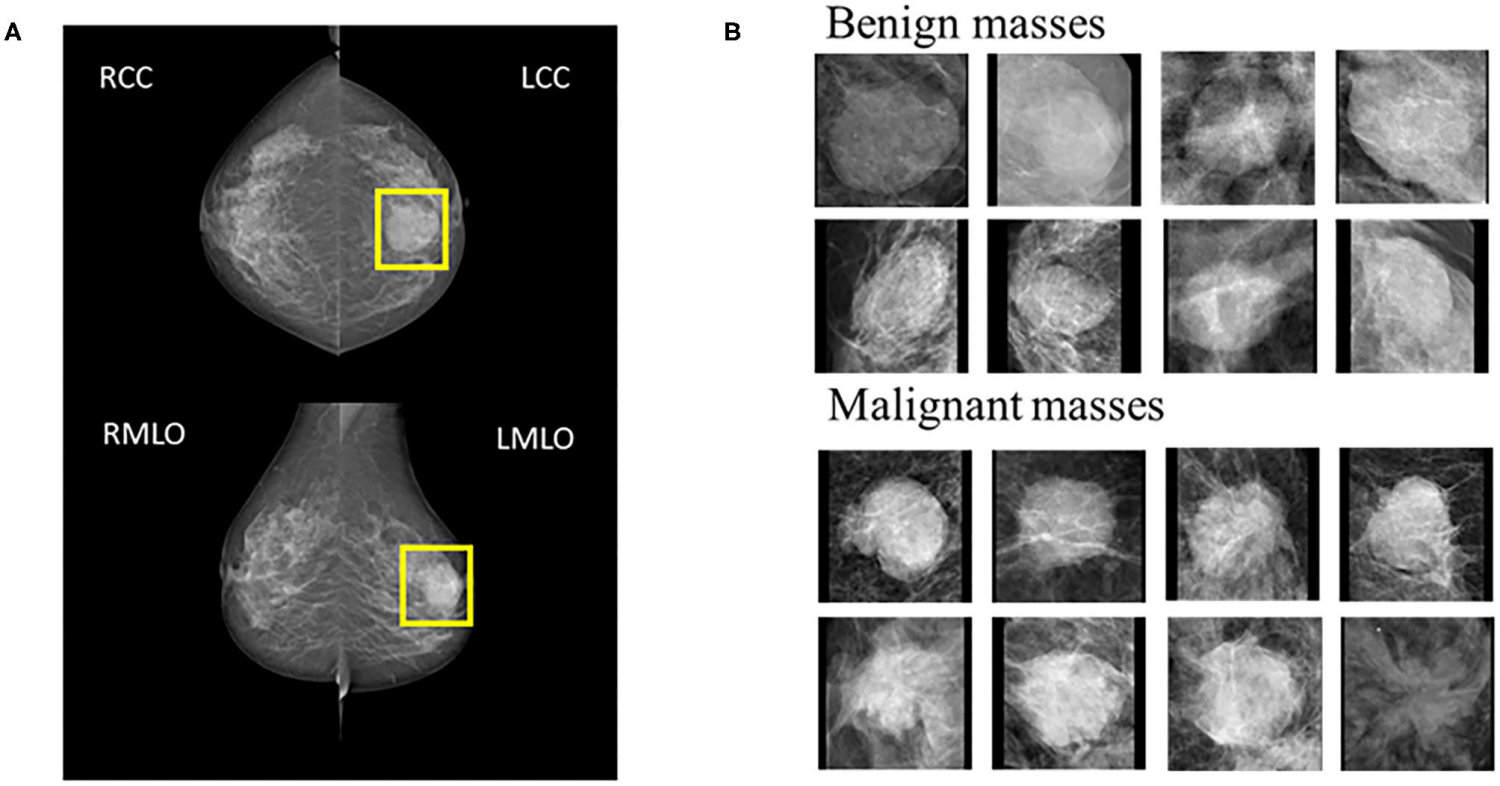
**(A)** Example cases on CC and MLO views. The yellow square represents the suspicious area labeled by the radiologist. **(B)** 8 benign and 8 malignant masses.

Mammography produces two images on both Cranio-Caudal (CC) views and the Medio-Lateral Oblique (MLO) (Figure 1). Five hundred and twenty-four patients were enrolled prospectively (confirmed by pathology) with digital mammography masses, including 988 mammography images (malignant: 494, benign: 494). Inclusion criteria: mammography images classified as Breast imaging reporting and data system (BI-RADS) 3, 4, and 5; BI-RADS 3 means probably benign, BI-RADS 4 means suspected malignancy, and BI-RADS 5 means highly suspected malignancy (20). Mass areas were labeled on MLO and CC views, respectively, in rectangular frames. Images were saved as 2-dimensional Digital Imaging and Communications in Medicine (DICOM) files with a 16-bit gray level.

### Data Preprocessing

All mammography images were preprocessed per the steps below:

Step 1: background removal. Regions of interest (ROIs) were obtained using a cropping operation on the mammography in order to remove the unnecessary black background.

Step 2: image normalization. ROIs were converted to a range [0, 1] with the linear function below (Func.1), which revealed that the original data were scaled in proportion. X_(norm) normalized data, X is the original data of mammography image, X_(max) and X_(min) are the maximum and minimum values of the original data, respectively.

X_(norm) = (X-X_(min))/(X_(max)-X_(min)) (Func.1).

Step 3: ROI size normalization. To meet standard input dimension requirements for most CNN, zero-filled images were achieved under no deformation conditions, and adjusted to 224 × 224 (The right of Figure 1).

Step 4: data sets separation. Training cohort (*n* = 744) and test cohort (*n* = 244) were created *via* random splitting. ROIs of test cohort were not enrolled into the training cohort.

Step 5: data augmentation. For each ROI in the training cohort, we used a combination of flipping and rotation transformations (90, 180, and 270 degrees), aiming at generating seven new label-preserving samples.

### Transfer-Learning and DL Fusion Network

**Figure 2:**
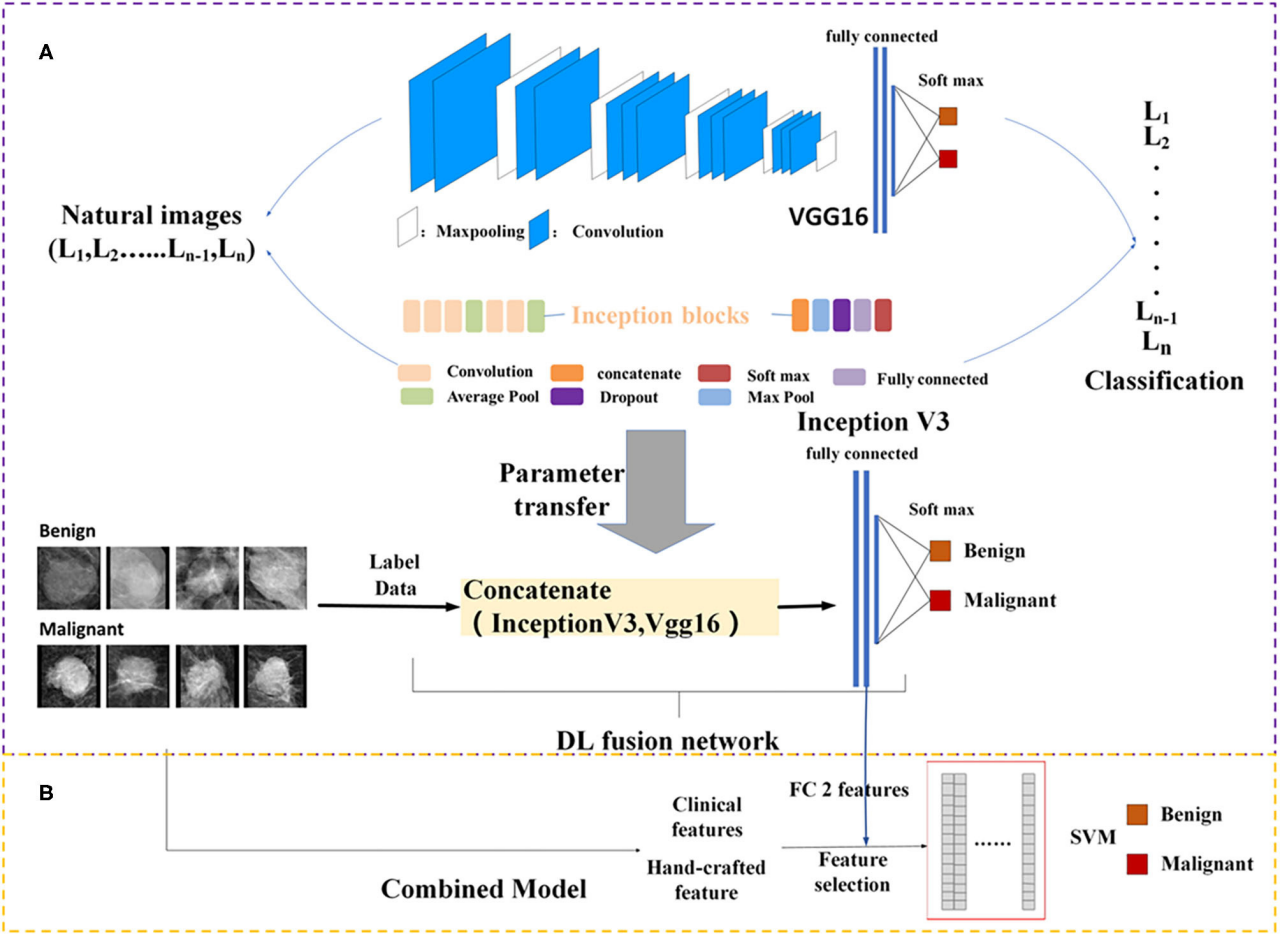
Framework of the proposed model structure. **(A)** DL fusion network, **(B)** combined model. FC, fully connected; SVM, Support Vector Machine; DL, deep learning.

DL architectures have three main components including convolutional layer, pooling layer, and fully connected (FC) layer. It is assumed that transfer of such sets with some fine tuning for the target network would be robust. Therefore, Vgg16 (21) network-based transfer-learning was used for this study. The VGG16 network has been pre-trained on the ImageNet dataset (22). Learned weights of the network gained during pre-training were applied to the target network. We proposed a DL fusion network combining the Vgg16 and Inception-V3 (23) networks based on transfer-learning aimed at strengthening the ability of transfer-learning. The learned weights of the network were transferred to the DL fusion network shown in Figure 2A. GlobalMaxPooling was used separately on the two networks to retain more information. The two networks were connected, and three FC layers were added to the fusion network. Additionally, the robustness of the DL fusion network was compared with that of the Vgg16 network.

The architecture of the Vgg16 fine-tuned network is shown in Table 1. The input layer of the image consisted of three parts: width, height, and channel. The input image size was 224 × 224 × 3. The number of layers after the first 12 layers were used for training. The epoch and the learning rate of the network were set to 200 and 1e-4, respectively. The Stochastic Gradient Descent (SGD) was used as the optimization algorithm (24). The momentum was set at 0.9, and the weight decay was set at 5 × 10^−4^. The fully connected layer was regularized using the dropout (25), with the last layer corresponding to the soft-max classifier. DL fusion network parameter settings referred to the VGG16 fine-tuned network. We performed a simulation of the python environment. The DL network training was performed on one GeForce GTX 1080Ti GPU.

**Table 1:** CNN network structure parameters.

**Vgg16 fine tuning**	**Out**
**learning layer type**	
Conv1-2	(224 × 224 × 64)
Max_Pooling	(112 × 112 × 64)
Conv3-4	(112 × 112 × 128)
Max_Pooling	(56 × 56 × 128)
Conv5-7	(56 × 56 × 256)
Max_Pooling	(28 × 28 × 256)
Conv8-10	(28 × 28 × 512)
Max_Pooling	(14 × 14 × 512)
Conv11-13	(14 × 14 × 512)
Max_Pooling	(7 × 7 × 512)
GAP	(1 × 1 × 512)
FC_1	(1 × 1 × 1024)
Dropout	(1 × 1 × 1024)
FC_2	(1 × 1 × 2)
Soft-max output	P

*Conv, convolutional layer; GAP, Global Average Pooling; FC, fully connected; P, Probability*.

### Feature Extraction

#### Handcrafted-Based Features

Texture contains important information from many types of images (26), and this information was used for classification and analysis. Four different types of hand-crafted features are extracted separately from first-order histogram features, second-order texture features, Hu's moment invariants features, and high-order Gabor features (a total of 455 features). The second-order texture features include gray-level co-occurrence matrix (GLCM), gray-gradient co-occurrence matrix (GLGCM), gray-level difference statistics (GLDS), gray run-length matrix (GLRLM), local binary pattern (LBP), and Gaussian Markov random field (GMRF) features. Hand-crafted feature extraction algorithms were implemented on MatLab 2018a.

#### Clinical Features

The clinical features of the patients are shown in Table 2. Morphological descriptions of mass are encoded as numerical values to obtain true feature values.

**Table 2:** Clinical features description of patients.

**Clinical features**	**Feature coding**
Shape	1-round, 2-oval, 3-irregular
Margin type	1-clear, 2-shadow, 3-differential leaf, 4-fuzzy, 5-glitch
Breast composition	1-The breasts are almost entirely fatty.
	2-There are scattered areas of fibro glandular density.
	3-The breasts are heterogeneously dense, which may obscure small masses.
	4-The breasts are extremely dense, which lowers the sensitivity of mammography.
Age	20–80 years old
Mass size	The diagonal pixel values of the ROI extracted can be roughly used as a method of measuring the size of the mass.

#### DL-Based Features

A trained DL model can be used as a feature extractor to extract features of different layers in the model. We proposed a DL fusion network to extract deep feature information on masses. The DL fusion network converts the image of the mass into a 1024-dimensional feature vector. In this study, we referred to this high dimensional vector from the second FC layer of the network as the DL feature.

### Feature Selection

Feature selection is another key step in radiomics, which means selecting a subset of relevant features based on the evaluation criterion. To reduce the training time of the model and improve its robustness and reliability, we used the minimal-redundancy-maximal-relevance (mRMR) (27) method to select the most significant feature sets. Through feature selection, 30 hand-crafted features (shown in Table 3) and 27 DL features were selected for input into the classifier.

**Table 3:** Hand crafted-based radiomics features after feature selection.

**Texture type**	**Texture descriptors**	**Number of**
		**features selected**
Second-order	Gray gradient co-occurrence	1
texture features	matrix features	
	Gray run-length matrix	2
	Gaussian Markov random field	4
	Gray-level difference statistics	1
	Local binary pattern	1
Higher-order	Gabor features	21
features		

### Model Construction

Because clinical features and mammography imaging express different types of information of a mass, we combined two types of information for exploratory analysis. Image biomarkers and clinical parameters were then processed using Min-Max normalization (as shown in Figure 2B). The support vector machine (SVM) (28, 29) with a linear kernel was used in the classification of breast masses. The SVM model aims to provide an efficient calculation method of learning by separating hyperplanes in a high dimensional feature space. A systematic review of machine learning techniques revealed that the SVM model is widely applied in breast tissue classification (30). In this study, the SVM hyper-parameters were fine-tuned through an internal grid search with 10-fold cross-validation.

### Training, Testing, and External Validation

We trained the proposed model using data (image biomarkers and clinical biomarkers) from the training set (744 ROIs). The prediction performance and model stability of the clinical model and the combined model were evaluated in the test set (244 ROIs) and verified in the external validation set (100 ROIs from 58 patients). The 58 patients in the external validation set came from the Yantai Yuhuangding Hospital.

### Evaluating Predictive Performance

Verifying the stability of the generated model using corresponding evaluation indicators is a key step to evaluating predictive performance. We established a confusion matrix to evaluate the proposed approach. We calculated the AUC (areas under the curve), accuracy, sensitivity, specificity, precision, and F_score from the confusion matrix to estimate the discriminant performance and stability of these models. Delong's test (31) was performed to evaluate the statistical significance of the AUC of the results. *P* < 0.05 was considered significant.

## Results

**Figure 3:**
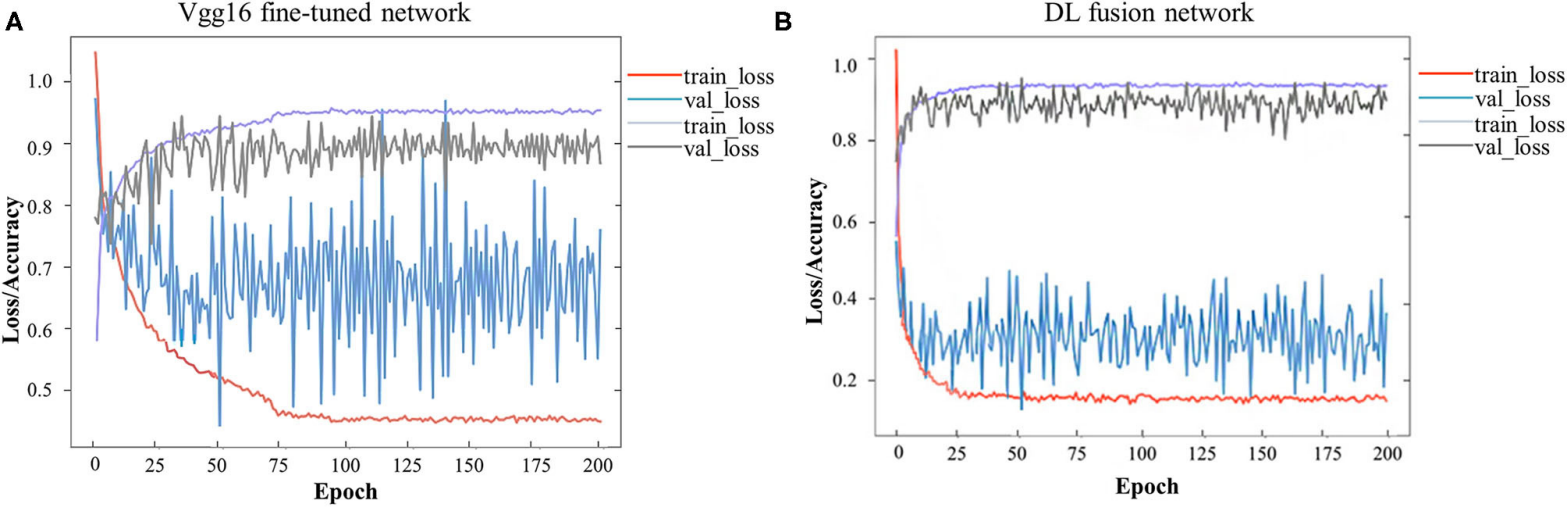
Loss and accuracy over epochs of training/ validation process. **(A)** Vgg16 fine-tuned network, **(B)** DL fusion network.

A total of 744 and 244 ROIs were randomly selected for the training cohort and test cohort, respectively. Figure 3 presents the convergence process and the training result of the Vgg16 fine-tuned network and the DL fusion network. The loss of the Vgg16 fine-tuned network fluctuated considerably for the worse convergence. The DL fusion network yielded better performances, as illustrated in Table 4. The accuracy of the DL fusion network improved to 87.30%, a 0.83% increase, compared to the Vgg16 fine-tuned network.

**Table 4:** Classification performance of Vgg16 fine-tuned network and DL fusion network.

**Trained networks**	**Sensitivity (%)**	**Specificity (%)**	**Accuracy (%)**	**F-score**
Vgg16 fine-tuned	82.79	90.16	86.47	85.96
network				
DL fusion	84.43	90.16	87.30	86.92
network				

**Figure 4:**
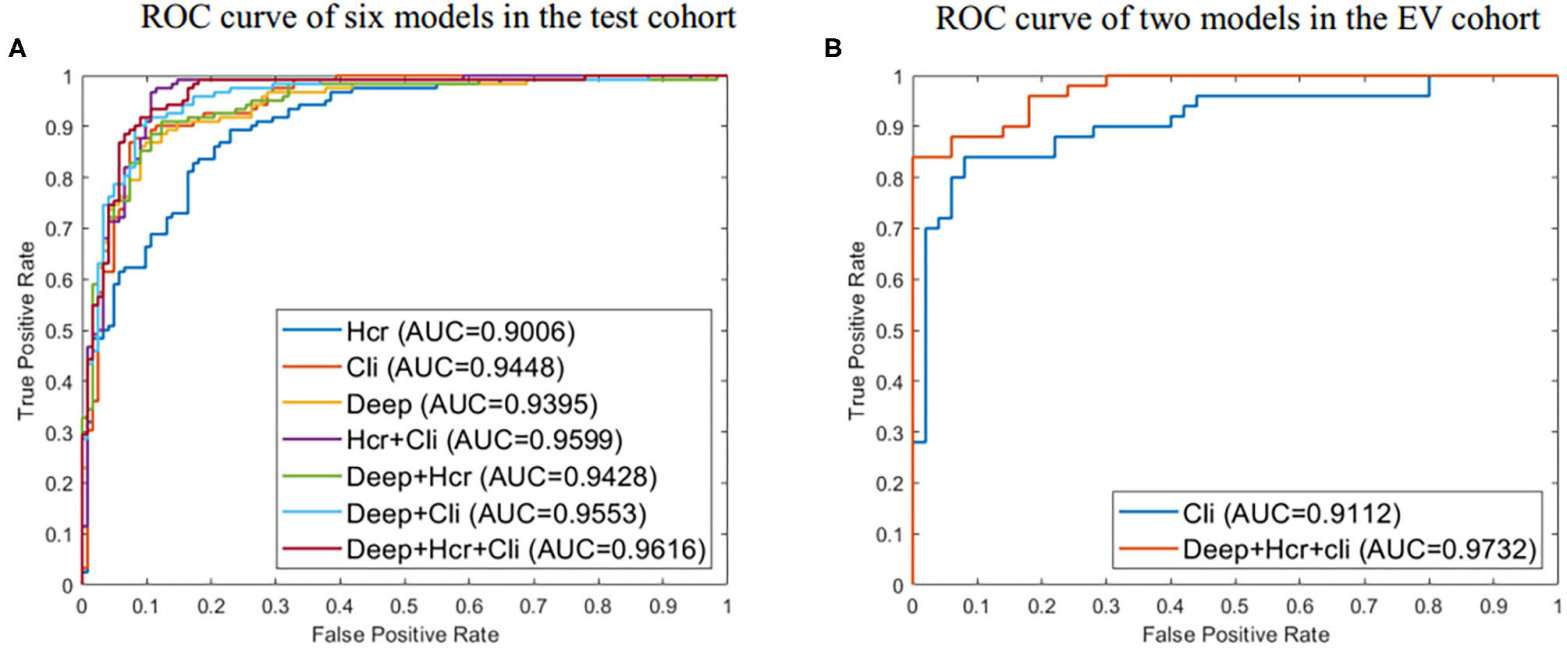
**(A)** ROC curve for evaluated predictive performance of seven methods in test cohort. **(B)** ROC curve for evaluated predictive performance of the external validation (EV) set. Deep represents 27 deep learning (DL) features. Hcr, hand crafted-based radiomics features; Cli, Clinical.

Using the mRMR feature selection method, 30 hand-crafted features (9 texture features, 21 higher-order features) and 27 DL features (1024 reduced to 27 dimensions) were selected (Table 3). A comparative view of seven feature combination schemes used for the classification of SVM is illustrated in Table 5, while the ROC curves for the evaluated representations of the seven schemes in the test cohort are shown in Figure 4A.

**Table 5:** Classification performance of different feature combination schemes in test cohort and validation cohort.

**Feature combination schemes**	**Sp (%)**	**Acc (%)**	**Sn (%)**	**Pre (%)**	**F-score (%)**	**AUC**
Hcr	83.61	79.92	76.23	82.30	79.15	90.06
Clinical	88.52	88.93	89.34	89.61	88.98	94.48
Hcr+Clinical	90.16	89.34	88.52	90.00	89.26	95.99
Deep 27	90.16	88.11	86.07	89.74	87.87	93.95
Deep 27+Hcr	90.98	87.70	84.43	90.35	87.29	94.28
Deep 27+Clinical	91.45	89.75	87.70	91.80	89.54	95.53
Deep 27+Clinical+Hcr	93.44	91.00	88.53	93.10	90.76	96.16
clinical model	94.00	83.00	72.00	92.31	80.90	91.12
combined model	100	90.00	80.00	100	88.89	97.32

*Deep 27, 27 deep learning (DL) features; Sp, specificity; Acc, accuracy; Sn, Sensitivity; Pre, Precision; Hcr, hand crafted-based radiomics features; AUC, area under the receiver operating characteristic curve. Clinical model and combined model are validated in the validation cohort (n = 100)*.

In the test cohort, the clinical model attained a classification accuracy of 0.889, a specificity of 0.885, and an AUC of 0.944. Compared with clinical models or other models, the progress made by the combined model in discriminative performance was more significant (accuracy = 0.910, specificity = 0.934, AUC = 0.962). The accuracy of the combined model rose by 3–11%, compared to models with a standalone image feature.

In the external validation set, the combined model was also proven to have better robustness and reliability (Table 5). The combined model yielded an improved accuracy and AUC, compared to the clinical model (accuracy: 0.900 vs. 0.830; specificity: 1.000 vs. 0.940; AUC: 0.973 vs. 0.911; *P* = 0.019). The ROC curves of the two models in the external validation cohort are shown in Figure 4B.

## Discussion

We conducted this study to develop an approach that combines radiomics features (Handcrafted-based and deep learning-based features) with clinical parameters for the assessment of the effects of classification in clinical practice. We also sought to reveal the classification performances of both the combined model and clinical parameters. As a result, we demonstrated the significance of combining image biomarkers with clinical parameters. Additionally, we observed a significant difference between the combined model and the clinical model; the former was more robust than the latter.

Interpreting the prediction performance of the combined model is not easy and must be done with caution to avoid drawing shallow conclusions. As shown by our results, the predictive performance improved when clinical data were added. The results of the combined model were improvements on those of the clinical model or other models, as illustrated in Table 5. Moreover, in the external validation cohort, the prediction performances of the combined model for benign and malignant masses were better than those for the clinical model (accuracy: 0.900 vs. 0.830; specificity: 1.000 vs. 0.940; AUC: 0.973 vs. 0.911; *P* = 0.019). There are potentially two major reasons for this outcome: first, the DL network design. The DL fusion network tries to encode breast mass images into deep features reflecting the internal information of masses. The neural network extracted abstract and complex features from the convolutional layers to the FC layers; second, clinical parameters are descriptive and distinguishable as a reference for BIRADS classification, which makes the results acceptable. But, because of the heterogeneity of breast masses, clinical parameters can indicate only limited mass information; the combined model carries information on intra-tumor heterogeneity, capturing the spatial relationships between neighboring pixels. Thus, performance largely depends on the ability of image biomarkers to distinguish between benign and malignant lesions.

Past studies have documented radiomics features' representation of valuable information from mass images (9–11). Radiomics features have been widely identified as reliable and useful biomarkers in clinical practice (8). The final goal of this extraction method is to generate image biomarkers to build a model for the improvement of clinical decisions. With the shift from hand-crafted to DL-based radiomics, combining deep learning features with hand-crafted features has become a popular approach in radiomics most recently (18, 19). The importance of clinical parameters has been reported in an experimental study by Moura et al. (32). Our current findings are based on expanding these results and prior works. To do this, we quantified the characteristics of mass imaging from many aspects using data-characterization algorithms. We extracted five clinical parameters, 1024 DL, and 455 hand-crafted features from each ROI. For deep learning feature extraction, we established the DL fusion network by transfer-learning. We trained the network through a patch-based strategy. In the past, superior performances have been achieved in a pre-trained network, compared to training from scratch (33), primarily because network training from scratch is too complicated and prone to over-fitting for small datasets (34). Hand-crafted features likely played a role in texture characterization. Redundant features were removed using mRMR, and features that can reflect the essential meaningful features of masses were retained. The SVM classification method was also chosen for comparative analysis.

Our research had three main advantages vis-à-vis previous studies using radiomics (18, 32, 33). First, we used a DL fusion network for feature extraction. DL fusion network can learn the intrinsic characteristics of mass images automatically from imaging data. Therefore, the DL fusion network does not need hand-coded feature extraction. Second, we combined image biomarkers with clinical parameters to assess the effects of the classification in clinical practice. Finally, we used an external validation set, which allowed us to extend the experimental results to other institutions and environments, providing more credibility to our inference.

Despite the promising outcome of this investigation, we had some limitations. The specific characteristic difference between the convolutional layer and the FC layer was not explored. Furthermore, because of the few medical image datasets, the model validation cohort in this study did not reach an optimal level. In future work, we intend to use more samples from other publicly available datasets, such as Mammographic Image Analysis Society (MIAS) and Database for Screening Mammography (DDSM) datasets. This will provide data diversity in terms of feature representation and may also improve overall architecture and network performance. Additionally, we plan to explore the predictive performance of different layer features.

In conclusion, combining radiomics features with clinical parameters can potentially serve a role in the prediction of benign or malignant breast masses. Additionally, this combination has stronger prediction performance, compared with clinical parameters. This study, therefore, developed a strategy that combines deep learning with traditional machine learning approaches to assist radiologists in interpreting breast images.

## Data Availability Statement

The raw data supporting the conclusions of this article will be made available by the authors, without undue reservation.

## Author Contributions

JZ, YC, and JD conceived and designed the experiments. YC performed the experiments. JZ, YL, TB, and DX analyzed the data. YC, JZ, and YL participated in writing manuscript. The final version of the manuscript has been reviewed and approved for publication by all author.

## Conflict of Interest

The authors declare that the research was conducted in the absence of any commercial or financial relationships that could be construed as a potential conflict of interest.
