# Erratum: Improving the Prediction of Benign or Malignant Breast Masses Using a Combination of Image Biomarkers and Clinical Parameters

**DOI:** 10.3389/fonc.2021.694094

**Published:** 2021-04-29

**Authors:** Frontiers Production Office

**Affiliations:** Frontiers Media SA, Lausanne, Switzerland

**Keywords:** mammography, image feature, deep learning, clinical prediction, radiomics

Due to a production error, in the original article, references (1–15) were incorrectly ordered. The correct order and references are provided below. The publisher apologizes for this error and state that this does not change the scientific conclusions of the article in any way. The original article has been updated.
